# Mutations in *STK11 *gene in Czech Peutz-Jeghers patients

**DOI:** 10.1186/1471-2350-10-69

**Published:** 2009-07-19

**Authors:** Peter Vasovčák, Alena Puchmajerová, Jan Roubalík, Anna Křepelová

**Affiliations:** 1 Department of Biology and Medical Genetics, Charles University 2nd Medical School and University Hospital Motol, Prague, Czech Republic; 2 Bata Hospital, Digestive Endoscopy Centre, Zlin, Czech Republic

## Abstract

**Background:**

Peutz-Jeghers syndrome (PJS) is an autosomal dominant hereditary disease characterized by mucocutaneous pigmentation and gastrointestinal hamartomatous polyposis. The germline mutations in the serine/threonine kinase 11 (*STK11*) gene have been shown to be associated with the disease. Individuals with PJS are at increased risk for development of various neoplasms. The aim of the present study was to characterize the genotype and phenotype of Czech patients with PJS.

**Methods:**

We examined genomic DNA of 8 individuals from five Czech families by sequencing analysis of *STK11 *gene, covering its promotor region, the entire coding region and the splice-site boundaries, and by multiplex ligation-dependent probe amplification (MLPA) assay designed for the identification of large exonic deletions or duplications of *STK11 *gene.

**Results:**

We found pathogenic mutations in *STK11 *gene in two families fulfilling the diagnostic criteria of PJS and in one of three sporadic cases not complying with the criteria. The patient with the frameshift mutation in *STK11 *gene developed aggressive gastric cancer. No other studied proband has developed a carcinoma so far.

**Conclusion:**

Our results showed that a germline mutation of *STK11 *gene can be found not only in probands fulfilling the PJS diagnostic criteria, but also in some sporadic cases not complying with the criteria. Moreover, we observed a new case of aggressive gastric cancer in a young patient with a frameshift mutation of *STK11 *gene.

## Background

Peutz-Jeghers syndrome (PJS; OMIM 175200) is an autosomal dominant disorder characterized by mucocutaneous pigmentation and gastrointestinal hamartomatous polyposis with an increased risk of cancer [1-4]. The cumulative risk of all cancers in PJS patients by the age of 60 years is 60% and is increased approximately by 8-fold as compared to general population [5]. Histopathologically, polyps in PJS are characterized as hamartomas. However, adenomatous changes may occur in polyps and they can become malignant. In addition to an elevated risk of gastrointestinal cancers, it has been described an increased risk of cancer development at other sites, particularly in the breast, ovary, uterus, cervix, pancreas, lung and testis [3,6-9]. Testicular sex cord and Sertoli cell tumors, leading to sexual precocity and gynecomastia [10-12], sex cord tumors with annular tubules and cervical adenoma malignum [13] have also been reported.

The gene responsible for PJS, denoted *STK11*, which encodes a serine/threonine kinase and mapps to chromosome 19p13.3, acts as a tumor suppressor [4,14,15]. It plays a role in the p53-dependent apoptosis pathway, in the vascular endothelial growth factor signaling pathway and in the polarization of epithelial cells [16-18].

About one-third of patients with PJS are diagnosed before the age of 10 years and up to 60% cases develop their first clinical manifestations until the third decade of life [19]. In most cases, initial symptoms are abdominal pain due to intussusceptions, obstruction and gastrointestinal bleeding with anemia [20,21]. A working definition of PJS has been suggested by Giardiello [3], where for individuals with a histopathologically confirmed hamartoma, the diagnosis of definite PJS requires two of the following three findings: a family history consistent with the autosomal dominant inheritance, mucocutaneous hyperpigmentation, or small-bowel polyposis. Tomlinson and Houlston [22] have modified the classification criteria for PJS for individuals without a family history of PJS, in whom the diagnosis depends on the presence of two or more histologically verified Peutz-Jeghers-type hamartomatous polyps.

There are some differential syndromes of PJS which could be misdiagnosed. The pigmentation of the perioral region is an external hallmark of PJS. It is not present in other hamartomatous polyposis syndromes which include Cowden syndrome (CS; OMIM 158350), Bannayan-Riley-Ruvalcaba syndrome (BRRS; OMIM 153480) and Juvenile polyposis syndrome (JPS; OMIM 174900). Laugier-Hunziker syndrome (LHS) is another differential diagnosis of PJS characterized by benign melanotic pigmentation of the oral cavity and lips, associated with spotted macular pigmentation of the fingerprints and longitudinal melanonychia. LHS is known to be a benign disease without gastrointestinal polyposis and with no systemic manifestation [23].

We report here a clinicopathological manifestation and mutational analysis of *STK11 *gene in eight PJS individuals from five unrelated Czech families.

## Methods

### Patients

Eight patients from five unrelated families were included in the study (table 1). Four probands from two families fulfilled and three sporadic cases did not fulfill criteria to establish the diagnosis of definite PJS [3,22]. In one individual, we made a presumptive diagnosis of PJS due to a first-degree relative with PJS and the presence of mucocutaneous hyperpigmentation. All eight patients except one (A-2) underwent endoscopic procedures to examine the inspectable part of GIT.

**Table 1:** Clinical manifestations

Family	Case no	Sex	Age at onset/admission	Initial symptoms/signs	Histology of polyps	Location	Cancer	Mutation
A	*A-1	F	10	pigmentation	hamartomatous	throughout GIT	lung, stomach	+
	A-2	F	2	pigmentation	NA	NA	No	+
B	*B-1	F	36	pigmentation	hamartomatous	throughout GIT	No	+
	*B-2	M	10	pigmentation	hamartomatous	throughout GIT	No	+
	*B-3	M	6	pigmentation	hamartomatous	throughout GIT	No	+
C	C-1	F	2	pigmentation	adenomatous	small intestine	No	+
D	D-2	M	50	pigmentation	hyperplastic	colon	No	-
E	E-3	M	10	pigmentation	no polyps	no polyps	No	-

NA – not analysed, GIT – gastrointestinal tract, * – fulfillment of PJS criteria

Family A includes mother (case A-1) and her daughter (case A-2).

Case A-1 was a 29-year-old female with negative family history. The diagnosis of PJS was made at her 10 years of age due to hyperpigmentation of the lips, buccal mucosa, and perinasal region. X-ray examination of abdomen did not reveal any polyp. The patient was free from any abdominal symptoms. At her 24 years of age she underwent gastroscopy because of dyspepsia lasting for a few months. A rigid mucosa of the stomach was noted, but it was histolopathologically negative. Sixteen months later and seven months after giving birth, two hamartomatous polyps 4.5 cm and 1.5 cm in diameter and multiple small polyps 1–3 mm in diameter were found in her stomach. Colonoscopy revealed tubulous adenoma, 3 cm in diameter in her caecum. Enteroclysis did not show any pathology of the small intestine. Later on, she has been followed up every six months. During the follow up, a tubulovillous adenoma from sigmoid colon and a hamartomatous polyp from the transverse colon were removed, and at her 27 years of age, a well differentiated mucinous adenocarcinoma in her left inferior lung lobe was surgically removed. One year later, during second gestation, adenocarcinoma of the stomach was found. The patient refused termination of her pregnancy. Therefore, an operation was performed without any previous neoadjuvant therapy. Unfortunately, because of deterioration of her performance state, premature birth was induced at the 30-th week of the gestation. Three months later the patient died of gastric cancer.

Case A-2, a 7-year-old girl, presented with pale brown patches on the lower lip, which have been noted since her 2 years of age. Examination of her GIT was not performed.

The younger daughter, 4 years old, was not included in the study. She was free of any symptoms typical for PJS.

Family B comprises mother (case B-1) and her two sons (cases B-2 and B-3).

Case B-1, a 46-year-old female has presented with perioral and buccal pigmentation, lasting since childhood. She was found to have colonic and small intestinal hamartomatous polyps already at 36 years of age. The polyps from the stomach were histopathologically classified as hyperplastic with diffuse mixed inflammatory infiltration in stroma and focal epithelial metaplasia. Subsequently, repeated colonoscopy and enteroscopy with polypectomy have been performed. Eventually, total colectomy was performed due to excessive polyposis and recurrent GIT problems. Histopatholological examination of the polyps did not reveal any malignancy. Her family history is missing.

Case B-2 represented a 17-year-old boy with perioral brown pigmentation, mostly on the lips. At his 10 years of age, hamartomatous polyps in small the intestine were detected. Afterwards, he has been frequently examined by gastroduodenoscopy and colonoscopy and polypectomies have been performed. One polyp from the antrum of the stomach, 3 mm in diameter, was classified as hyperplastic with stromal inflammation. Three colon polyps, 3 mm in diameter, showed the typical features of hamartomatous lesions, branching strands of muscular tissue and numerous cystic dilatations of the glandular lumens of various sizes.

Case B-3, a 13-year-old boy, manifested with mucocutaneous brown to dark blue pigmentations on the lip, mostly on the lower one. At his 6 years of age, rectal bleeding due to a polyp, 55 × 35 × 20 mm, in the rectum was noted. Histopathological examination of the polyp showed a tubulovillous adenoma with mild dysplasia in the superficial colonic epithelium. Later on, he has undergone frequent gastroduodenoscopies and colonoscopies with polypectomies. Three polyps were classified as hamartomatous and one as a tubulovillous adenoma with low-grade dysplasia, 3 mm in diameter. All four polyps were excised from the sigmoid colon.

The remaining cases (C-1, D-1 and E-1) were sporadic.

Case C-1, a 20-year-old female has presented brown to dark blue pigmentations since her 2 years of age. At her 14 years of age, enteroclysis her small intestine showed one adenomatous polyp. Frequent colonoscopies and enteroclysies with negative results have been performed. At the time of the molecular analysis of her genomic DNA for germline *STK11 *mutation, another capsule endoscopy examination was performed. A few (less than ten) diminutive polyps in the stomach and one polyp in the ileum were found. The polyp from the ileum showed characteristic histopathological features of adenoma. Polyps from the stomach were not biopsied. Her family history was negative.

Case D-1, a 50-year-old male with perioral and buccal pigmentation was found to have two hyperplastic polyps, one in the sigmoideum and the other one in the colon ascendens. Tubulovillous adenoma with low-grade dysplasia was excised from the ascending colon. The mother of the patient had colon cancer in 72 years of age and his father, a smoker, had lung cancer in 76 years of age. They were without hyperpigmentation.

Case E-1, a 10-year-old boy, was referred because of perioral and buccal pigmentation. Examination of his GIT did not reveal any polyp. His parents and step-siblings are without any PJS symptoms.

### Genetic analysis

After receiving a written and signed informed consent, the genomic DNA of the patients was isolated from blood leukocytes using the Genomic DNA Purification Kit (Gentra Systems, Minneapolis, MI, USA) according to manufacturer's guide. The genomic DNA was amplified using intronic primers [24,25] flanking the nine exons and the promoter region of *STK11 *gene. PCR reactions were performed in a total volume of 30 μl containing 100 ng of genomic DNA, 50 pmols of each primer, 0.2 mM of each dNTP, 1 × *Taq *buffer containing 1.5 mM MgCl_2 _and 1 unit of *Taq *polymerase (Fermentas, Vilnius, Lithuania). The amplification was performed in 2720 Thermal cycler (Applied Biosystems, Foster City, CA, USA) with the following protocol: an initial denaturation of 1 min at 95°C was followed by 33 cycles of 1 min at 95°C, 1 min at 53°C, 1 min at 72°C, and a final extension step of 7 min at 72°C. PCR products were purified using the SureClean PCR purification kit (Bioline, London, UK). DNA sequencing was performed using the purified PCR product, the BigDye Terminator v3.1 Cycle Sequencing kit and ABI 3130 Genetic Analyzer (both from Applied Biosystem) according to the manufacturer's instructions. Patients with negative results of DNA sequencing analysis were further examined by means of the multiplex ligation-dependent probe amplification (MLPA) method for the identification of large exonic deletions or duplications, using the P101-*STK11 *MLPA kit (MRC Holland, Amsterdam, The Netherlands) and the following MLPA protocol . Samples were run and data were analyzed on ABI 3130 Genetic Analyzer in conjunction with Genotyper software (version 4.0; Applied Biosystems). Electropherograms were evaluated by visual examination of peak heights of the *STK11 *fragments in relation to the adjacent control fragments and in comparison with external control DNA samples.

The study was conducted with approval by central ethical committee of Ministry of Health, Czech Republic, in accordance with the tenets of the Helsinki declaration.

## Results and discussion

**Figure 1:**
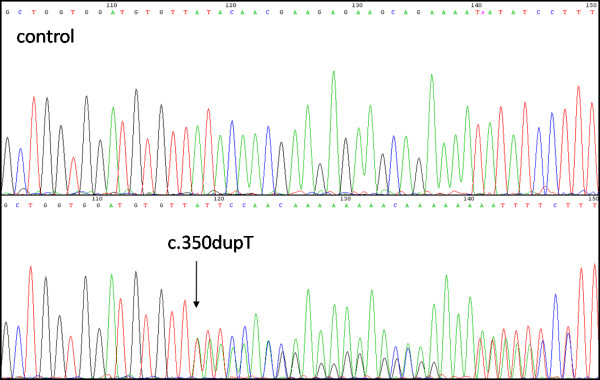
**Sequencing chromatogram from the analysis of *STK11 *gene in the family A showing a duplication of thymine in the position c.350, leading to a frameshift and a premature stop codon**.

Excised polyps were histopathologically classified as hamartomatous in the cases A-1, B-1, B-2, and B-3, as adenomatous in the case C-1, and as hyperplastic in the case D-1. Case E-1 was free of any polyps (Table 1). All studied individuals had pigmentation of the lips and buccal mucosa, while it was most visible in the children patients. None of the probands had pigmentation of extremities. A positive family history of cancer was only noted in the case D-1. Mutation analysis revealed three different germline mutations. In the family A, a germline mutation (c.350dupT) in exon 2 (Fig. 1) was detected. This mutation is predicted to introduce a frameshift at codon Leu117, 46 novel amino acid residues, and a premature termination codon (p.Leu117PhefsX46). It was found in heterozygosity in both examined patients (A-1 and A-2).

**Figure 2:**
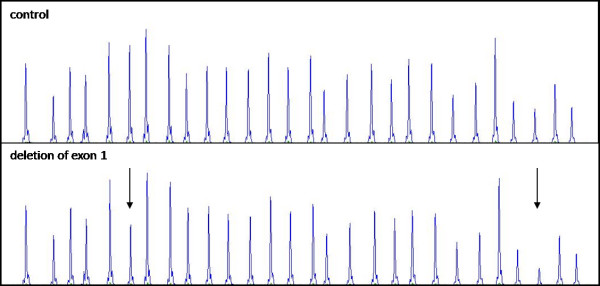
**Representative chromatogram from MLPA analysis of *STK11 *in the family B showing the relative reduction in the peak area of probes hybridising to exon 1 and a part of the promotor region (arrows mark the deleted regions)**.

**Figure 3:**
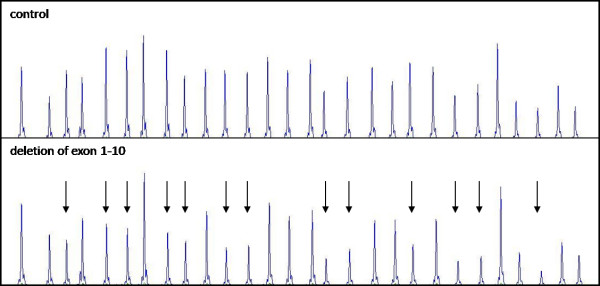
**Representative chromatogram from MLPA analysis of *STK11 *in the family C showing the relative reduction in the peak area of probes hybridising to exons 1–10 and the promotor region**.

Individuals from the family B (cases B-1, B-2, and B-3) harboured deletion of a part of the promoter region and exon 1 (Fig. 2). Case C-1 was a carrier of deletion of the whole *STK11 *gene (Fig. 3). In the cases D-1 and E-1, we have not revealed any variation of *STK11 *gene by using the aforementioned methods.

PJS is a relatively very well characterized disorder with a clear cut phenotype [22]. However, in sporadic cases, the diagnosis of PJS may be uncertain. Although multiple hamartomatous polyps of the GIT are pathognomonic of the PJS, hyperplastic and adenomatous polyps are commonly present [17]. Recently, it has been reported that *STK11 *deletions are not a rare cause of Peutz-Jeghers syndrome and account for up to 30% of patients with PJS [26,27]. There was no difference in the clinical phenotype between the patients with point mutations or with large genomic deletions [26]. However, the detailed phenotype of patients with different types of mutations was not reported.

Members of the family B (cases B-1, B-2, and B-3) had almost uniform clinical symptoms with variable age of onset of the first symptoms and detection of polyps. They are carriers of a germline mutation (deletion of a part of the promoter region and exon 1) of *STK11*. All three affected individuals had mucocutaneous hyperpigmentation predominantly on the lips and on the buccal mucosa, being the most prominent in the youngest patient (B-3) and very pale in the mother (B-1). Sons of the latter patient, i.e. the patients B-2 and B-3, were initially classified as PJS-suspected because of striking hyperpigmentation of the lips. Both of them are under careful surveillance. A few endoscopic examinations of the GIT have been performed so far, with polypectomy of hamartomatous polyps. This confirmed the diagnosis of definite PJS. Polyps were localized in the small intestine and colon. No polyps were detected in the stomach.

Deletion of the promoter region and exon 1 was reported in three independent studies in 12 PJS families overall [26-28]. It could be a recurrent mutation, probably a consequence of an unequal recombination mediated by repetitive Alu (SINE elements) sequences. In accord with UCSC Genome Browser, the region of chr19:1,129,999–1,196,665, where *STK11 *gene is located, is rich for these repetitive sequences, which can be involved in large chromosome rearrangements. The implication of Alu repetitive elements in unequal genomic recombinations were described for another tumor suppressor gene, *MSH2*, implicated in Lynch syndrome (HNPCC) [29].

We failed to find any variation of *STK11 *gene in sporadic cases D-1 and E-1, which would explain their phenotype. On the other hand, they did not fulfill criteria for the diagnosis of definite PJS [3,22]. We included these cases to the study on the basis of the result from the case C-1. Especially in case E-1 PJS polyps could develop later on. Studies with more individuals not fulfilling PJS diagnostic criteria were reported. None of the patients harboured a germline mutation of *STK11 *gene [26,30]. Some studies suggested there could be another locus responsible for PJS phenotype [31,32]. Other authors stated according to their results that another locus is unlikely and the causative variation could be in regulatory regions such as promoter, enhancers, or splicing sites deep in introns, which are not detectable by conventional methods [26,27,33].

The risk of developing various types of GIT cancers (in the esophagus, stomach, small bowel and colon) was determined in several studies [3,5,7,8,34]. The cumulative risk for stomach cancer was 29% [8]. Amos et al. noted that gastric polyps are very common among individuals with PJS [30]. However, they did not specify the proportion of patients with a detectable PJS germline mutation and the gastric polyps/cancer. There are several case-reports and reviews reporting gastric cancer in PJS patients [3,20,35-44]. In our group of probands, the case A-1 had developed gastric cancer at 28 years of age and died one year later. No genotype-phenotype correlations were published in PJS patients with gastric cancer [7,30,33]. Konishi et al. reviewed 103 PJS patients with malignancy from literature and found out that the mean age of 8 cases with gastric cancer was 31.2 years as compared to 39.7 years in duodenal carcinoma (9 cases), and 48 years in colorectal carcinoma (13 cases). According to the literature and our results we suppose that gastric cancer has very aggressive course in some individuals with PJS and despite the very frequent endoscopic examinations with relevant treatment the next course is usually poor. Therefore, more attention should be paid to patients with molecularly confirmed PJS, especially those who have polyposis of the stomach. It would be particularly interesting to find out if there is a correlation between the genotype and phenotype in relation to the development of gastric cancer. There have been only two reports dealing with gastric cancer in PJS patients and mutational analysis of *STK11 *gene so far [24,43]. Shinmura et al. described two PJS females (sisters) with gastric cancer in whom a *STK11 *germline mutation (c.890delG) was identified [24]. Takahashi et al. reported a 14-year-old girl with sporadic PJS and early-onset gastric cancer harboring a frameshift (c.757_758insT) *STK11 *mutation [43]. Similarly, as in our family A, the mutations led to a truncated protein lacking the kinase domain. These results suggest that the truncation mutations leading to loss of *STK11 *kinase domain could act in a dominant negative fashion and be responsible of tumor development. Schumacher et al. summarized clinical and mutational data from 132 PJS cases (83 without and 49 with cancer) to find correlation between the type/site of mutation and cancer. They proposed two different mechanisms of tumor development. One is based on the loss of *STK11 *functions due to truncation mutations and subsequent LOH as a second hit. This hypothesis is not in accord with findings of other authors, where a second hit was not a requisite condition for tumor development [24,43].

## Conclusion

In summary, we found germline mutations of *STK11 *gene in three families. One patient (C-1) with the germline mutation did not fulfill the criteria for establishing the diagnosis of PJS. Therefore, the variability in time of onset of symptoms should be always kept in mind when establishing the diagnosis of PJS and managing this disease.

## Abbreviations

*STK11*: serine/threonine kinase 11; PJS: Peutz-Jeghers syndrome; MLPA: multiplex ligation-dependent probe amplification; BRRS: Bannayan-Riley-Ruvalcaba syndrome; JPS: Juvenile polyposis syndrome; LHS: Laugier-Hunziker syndrome; GIT: gastrointestinal tract; SINE: short interspersed element; UCSC: University of California Santa Cruz; *MSH2*: mutS homologue 2; HNPCC: hereditary nonpolyposis colon cancer; LOH: loss of heterozygosity.

## Competing interests

The authors declare that they have no competing interests.

## Authors' contributions

PV carried out molecular genetic studies including DNA sequencing, MLPA analysis for all the families, and drafted the manuscript. AP identified and diagnosed the patients. JR performed GIT examinations and provided histopathological information. AK designed the study and revised the manuscript. All authors read and approved the final manuscript.

## Pre-publication history

The pre-publication history for this paper can be accessed here:


